# Dual Action of Lysophosphatidate-Functionalised Titanium: Interactions with Human (MG63) Osteoblasts and Methicillin Resistant *Staphylococcus aureus*

**DOI:** 10.1371/journal.pone.0143509

**Published:** 2015-11-25

**Authors:** Mette Elena Skindersoe, Karen A. Krogfelt, Ashley Blom, Guowei Jiang, Glenn D. Prestwich, Jason Peter Mansell

**Affiliations:** 1 Department of Systems Biology, Technical University of Denmark, Kgs. Lyngby, Denmark; 2 Department for Infection and Microbiology Control, Statens Serum Institut, Copenhagen S, Denmark; 3 Musculoskeletal Research Unit, University of Bristol, Southmead Hospital, Bristol, BS10 5NB, United Kingdom; 4 Department of Medicinal Chemistry, The University of Utah, 419 Wakara Way, Suite 205, Salt Lake City, Utah 84108, United States of America; 5 Department of Biological, Biomedical & Analytical Sciences, University of the West of England, Frenchay Campus, Bristol, BS16 1QY, United Kingdom; Université de Technologie de Compiègne, FRANCE

## Abstract

Titanium (Ti) is a widely used material for surgical implants; total joint replacements (TJRs), screws and plates for fixing bones and dental implants are forged from Ti. Whilst Ti integrates well into host tissue approximately 10% of TJRs will fail in the lifetime of the patient through a process known as aseptic loosening. These failures necessitate revision arthroplasties which are more complicated and costly than the initial procedure. Finding ways of enhancing early (osseo)integration of TJRs is therefore highly desirable and continues to represent a research priority in current biomaterial design. One way of realising improvements in implant quality is to coat the Ti surface with small biological agents known to support human osteoblast formation and maturation at Ti surfaces. Lysophosphatidic acid (LPA) and certain LPA analogues offer potential solutions as Ti coatings in reducing aseptic loosening. Herein we present evidence for the successful bio-functionalisation of Ti using LPA. This modified Ti surface heightened the maturation of human osteoblasts, as supported by increased expression of alkaline phosphatase. These functionalised surfaces also deterred the attachment and growth of *Staphylococcus aureus*, a bacterium often associated with implant failures through sepsis. Collectively we provide evidence for the fabrication of a dual-action Ti surface finish, a highly desirable feature towards the development of next-generation implantable devices.

## Introduction

Developing surface finishes to encourage osteoblastogenesis is a continuing theme of contemporary bone biomaterials research. Enhancing human osteoblast (hOB) formation and maturation at prosthetic surfaces is predicted to secure superior implant integration and longevity. Novel ways of fabricating unique substrates includes coating bone biomaterials with small biological agents. Importantly the selected molecules should target hOBs and their bone marrow-derived stromal cell (BMSC) precursors. Particularly attractive are agents that participate in signalling co-operation of “cross-coupling” with key molecules central to bone formation, health and homeostasis. Candidate factors fulfilling these criteria are the simple bioactive lysophosphatidic acids (LPAs) and/or their more stable, phosphatase-resistant analogues. The term LPA (1-acyl-2-hydroxy-*sn*-glycero-3-phosphate) actually refers to a group of lysophospholipids that are characterized as having a fatty acyl chain, glycerol backbone and a phosphate head group, different LPAs vary according to chain length and degree of saturation. The different LPAs interact with a wide range of G protein-coupled receptors (GCPR), and members of the LPA family are involved in diverse physiological activities such as wound healing, cell survival, apoptosis, motility and differentiation (reviewed in [1]). Skeletal cells, including hOBs and BMSCs, are also targets for LPAs [2]. Of particular relevance to bone formation is the discovery that LPA co-operates with the active metabolite of vitamin D_3_, 1,25-dihydroxy vitamin D_3_ (1,25D), to secure hOB maturation [3–5]. It is the mature osteoblast phenotype that is responsible for the provision of a mechanically robust, mineralised matrix. The signalling cross-coupling that occurs between LPA and active vitamin D_3_ metabolites culminates in synergistic increases in alkaline phosphatase (ALP), an enzyme absolutely essential for bone matrix calcification [6]. These features of LPA, its small size and ability to co-operate with 1,25D make it an especially desirable molecule for the functionalisation of titanium and hydroxyapatite, two widely used bone biomaterials. The only other molecules reported to co-operate with 1,25D in stimulating hOB maturation are TGF-β [7] and epidermal growth factor [8]. Their larger size and greater cost make them less desirable contenders for biomaterial conjugation and they are less likely to withstand conventional sterilisation protocols.

In addition to its reported effects on hOBs, 16:0 MPPA, an LPA with an unsaturated C16 chain, has been reported to inhibit virulence factor production and biofilm formation of the human opportunistic pathogen *Pseudomonas aeruginosa* [9]. 16:0 MPPA has together with several other phospholipids such as dipalmitoyl phosphatidyl serine, dipalmitoyl phosphatidic acid and monomyristoyl phosphatidic acid been shown to sensitize otherwise resistant *P*. *aeruginosa* isolates to the actions of betalactams [10]. We have also shown that 16:0 MPPA and monopalmitoyl phosphatidyl choline are antimicrobial against a range of gram positive bacteria such as *Staphylococcus aureus* and *Enterococcus* spp [11]. Collectively LPA-functionalised devices could be beneficial in two important ways; the enhancement of early osseointegration by promoting hOB maturation at the surface and secondly, a substrate that is less appealing to bacteria at the time of implantation.

Biological coating of contemporary bone biomaterials can be particularly challenging, often requiring time consuming, complex and costly procedures. However there is an attractive, facile method for functionalising titanium devices; the use of alkane phosphonic acids (APAs) which have a natural, high affinity for metal oxides [12] including titania (TiO_2_), the natural coating of titanium. The bonds formed between TiO_2_ and APAs are robust, iono-covalent in nature and are superior to those formed using silanes as no further cross-linking is required. The nature of the APA-TiO_2_ bond has been reported to be mono and/or bidentate and can also include electrostatic interactions [13,14].

Herein we report the facile functionalisation of Ti using octadecylphosphonic acid (ODPA) for the subsequent attachment of 16:0 MPPA and a palmitoyl, phosphatase-resistant LPA analogue, O-2-(hexadecyloxy)-3-methoxypropyl O-hydrogenphosphorothioate (16:0 OMPT) [15]. The rationale for using a phosphatase-resistant LPA species over 16:0 MPPA is to produce a Ti surface finish with increased “residence time” of the tethered bioactive as native LPAs are susceptible to rapid hydrolysis by lipid phosphate phosphatases [16]. These modified surfaces were evaluated for their ability to support 1,25D-induced hOB maturation. In parallel the same surfaces were exposed to *S*. *aureus* to investigate the effect of these functionalised materials on bacterial attachment and growth.

## Materials and Methods

### General

**Fig 1:**
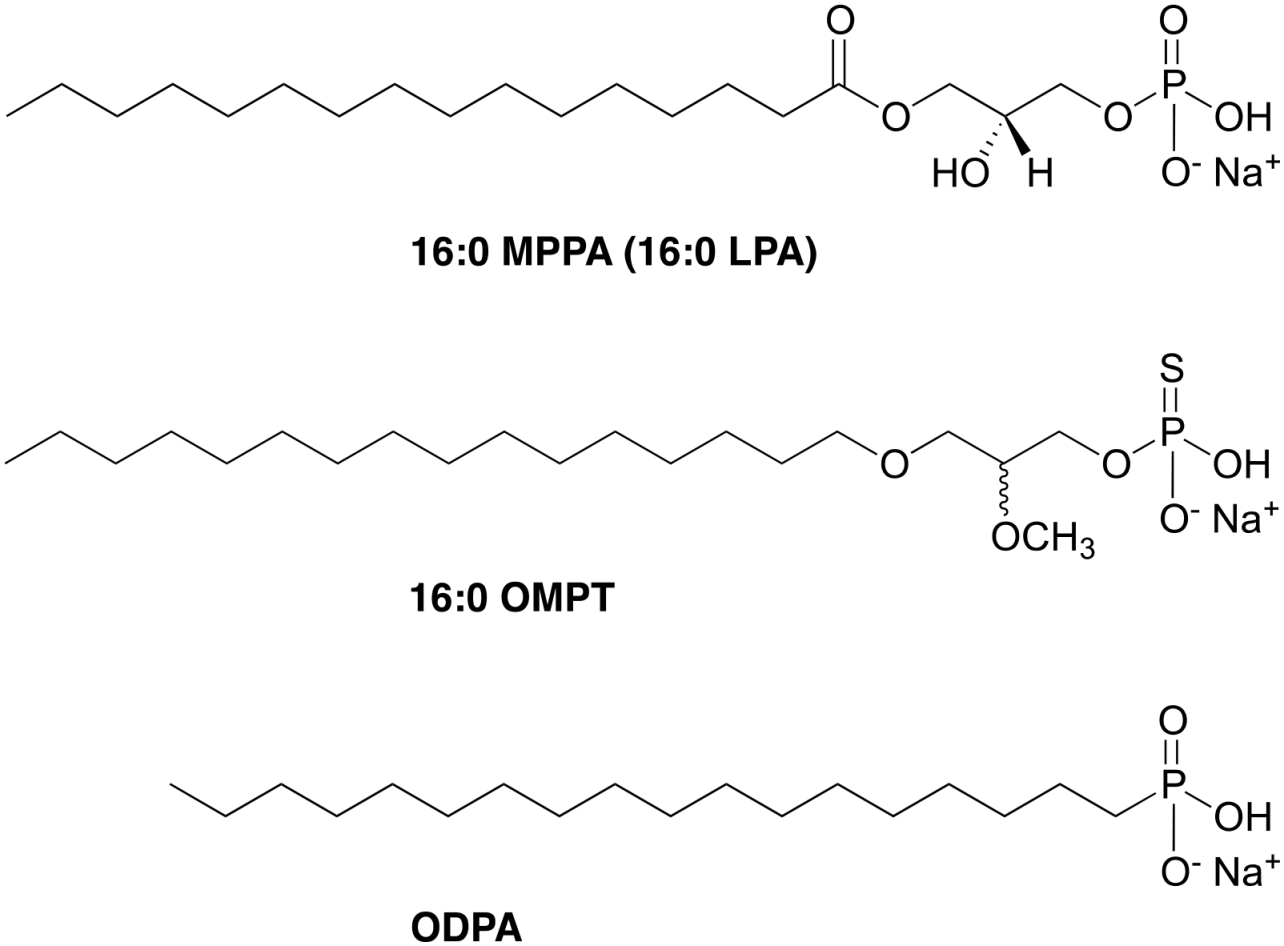
Structures of 16:0 MPPA, 16:0 OMPT and octadecylphosphonic acid (ODPA). The alkane phosphonic acid, ODPA, was used as a tether for the subsequent functionalisation of titanium with 16:0 OMPT, a phosphatase-resistant LPA receptor agonist.

Unless stated otherwise, all reagents were of analytical grade from Sigma-Aldrich (Poole, UK). Stocks of 16:0 MPPA (Cambridge Bioscience, Cambridge, UK) were prepared in 1:1 ethanol:tissue culture grade water to a final concentration of 10 mM and stored at -20°C. Likewise, 1,25D (100 micromolar) was prepared in ethanol and stored at -20°C. Stable, phosphatase-resistant 16:0 OMPT (Fig 1) was synthesized at The University of Utah as detailed below. The compound was reconstituted in 1:1 ethanol:tissue culture grade water to a final concentration of 10 mM and stored at -20°C. Octadecylphosphonic acid (ODPA) was dissolved in anhydrous tetrahydrofuran to a final concentration of 1 mM in glass and stored at ambient temperature.

### Synthesis of O-2-(hexadecyloxy)-3-methoxypropyl O-hydrogenphosphorothioate (16:0 OMPT)

#### 2-(hexadecyloxy)-3-methoxypropan-1-ol

A solution of 1-trityl-3-methyl-glycerol (2.537 g, 7.29 mmole) in 40 ml of dimethylformamide (DMF) was added sodium hydride (60% in mineral oil, 335 mg, and 1.15 eq. 8.39 mmole). Then the reaction mixture was stirred at room temperature for 1 hour before 1-bromohexadecane (2.67 g, 8.75 mmole, 1.2 eq.) in 10 mL DMF and tributylammonia iodide (0.1 eq, 0.73 mmole, 269 mg) were added. The mixture was stirred overnight at room temperature. Then 1N HCl (60 mL) was added to quench the reaction, and the mixture was extracted with 100 mL EtOAc twice. The organic extract was washed with saturated NaHCO_3_ solution, followed by a saturated NaCl solution. Solvent was removed at reduced pressure and the crude mixture was purified on silica column (EtOAc:hexane 5/95) to give crude hexadecyl ether (1.537 g, 2.67 mmole, 37%), which was directly dissolved into 30 mL dichloromethane and then 3 mL trifluoroacetic acid was added. The reaction mixture was stirred for 1 hour at room temperature. Then solid sodium bicarbonate was added slowly to neutralize the reaction mixture, followed by 100 mL of water. The organic layer was separated and the water layer was extracted by dichloromethane (100 mL) twice. All organic layers were combined and dried over sodium sulphate. The organic solvent was concentrated and the residue was purified on silica column (EtOAc:hexane 5/95, 15/85) to give 2-(hexadecyloxy)-3-methoxypropan-1-ol (722 mg, 2.19 mmole, 82%) as a waxy solid.

^1^HNMR (CDCl_3_) 3.70–3.75 (m, 1H), 3.55–3.65 (m, 2H), 3.42–3.52 (m, 4H), 3.34 (s, 3H), 1.51–1.58 (m, 2H), 1.20–1.38 (m, 26H).

#### O,O-Bis(2-cyanoethyl) O-2-(hexadecyloxy)-3-methoxypropylphosphorothioate

Di(2-cyanoethyl) diisopropylphosphorodiamidite (271 mg, 1 mmole) in 2 mL dichloromethane was added under an argon atmosphere to a solution of the above hexadecyl ether (330 mg 1 mmole) and 1*H*-tetrazole (70 mg 1 mmole) in 10 mL dry dichloromethane. After stirring for 1 hour, sulfur (32 mg, 1 mmole) and CS_2_/pyridine (0.2 mL, 1:1 v/v) were added. After stirring at room temperature for another 2 hours, the reaction mixture was filtered and filtrate was washed with brine, dried over sodium sulphate, and concentrated. The residue was purified on silica column (EtOAc:hexane 1:1, 1:2) to give O,O-Bis(2-cyanoethyl) O-2-(hexadecyloxy)-3-methoxypropylphosphorothioate (280 mg, 0.53 mmole, 53%) as a waxy solid.

^1^HNMR (CDCl_3_) 4.21–4.35 (m, 5H), 4.08–4.12 (m, 1H), 3.51–3.62 (m, 3H), 3.46–3.48 (m, 2H), 3.37 (s, 3H), 2.78 (t, J = 6.0 Hz, 4H), 1.51–1.71 (m, 2H), 1.20–1.38 (m, 26H), 0.88 (t, J = 6.8 Hz, 3H); ^31^P NMR (CDCl_3_) 68.8.

#### Sodium O-2-(hexadecyloxy)-3-methoxypropyl O-hydrogenphosphorothioate (“16:0 OMPT”)

Tert-butylamine (0.2 mL) was added to a solution of the above *O*,*O*-bis(2-cyanoethyl) O-2-(hexadecyloxy)-3-methoxypropylphosphorothioate (150 mg, 0.27 mmole) in CH_3_CN (1 mL) under N_2_ followed by the addition of bistrimethylsilylacetamide (0.2 mL). After 48 hours, the reaction mixture was concentrated and the residue was purified on silica column (EtOAc:CH_3_OH, 9:1) to afford 91 mg of the ammonium salt of the 16:0 analogue of OMPT as a light yellow oil (0.21 mmole, 78%). The monosodium salt was obtained via ion exchange resin (Dowex 200 Na^+^ form).

^1^HNMR (CDCl_3_) 4.05–4.22 m, 2H), 3.41–3.75 (m, 5H), 3.37 (s, 3H), 1.51–1.71 (m, 2H), 1.20–1.38 (m, 26H), 0.88 (t, J = 7.2 Hz, 3H); ^31^P NMR (CDCl_3_) 52.7. HRMS (MALDI) for C_20_H_42_NaO_5_PS [M^+^ +1]: found 448.9057, calculated 448.2388.

### Lysophosphatidate-functionalisation of Ti specimens

Titanium (Ti) discs (12.7 mm diameter and 2.5 mm thickness) of orthopaedic grade titanium were a generous gift from Corin (Cirencester, UK). To encourage oxide formation Ti discs were either oven baked at 160°C overnight (~18 hours) or treated with Piranha solution as described by us previously [17]. Briefly, Piranha solution was prepared by combining equal volumes (20 mL) of ice-cold concentrated sulphuric acid and 30% (w/v) hydrogen peroxide. [Please note that Piranha solution is highly corrosive and oxidative and should be handled with care]. Once mixed the resultant solution was allowed to reach room temperature before use. Ti discs were steeped in Piranha solution for 2 hours under gentle stirring at ambient temperature. Discs were subsequently washed and patted dry. Oven-baked Ti, Piranha-treated Ti and un-pre-treated Ti discs were then immersed in 1 mM ODPA in tetrahydrofuran (THF) for 1 hour at ambient temperature. The Ti specimens were allowed to dry in a fume cupboard at ambient temperature, and then given two 5-minute rinses in THF, allowed to dry followed by baking overnight at 160°C. Each Ti disc was then placed into a well of a 24-well tissue culture plate and bathed in 1 ml of 100 micromolar 16:0 OMPT in 50% aqueous (tissue culture grade water) ethanol, and samples left overnight at ambient temperature. Then the discs were rinsed in tissue culture-grade water and allowed to dry, at ambient temperature in a tissue-culture cabinet.

### Human MG63 osteoblasts

Human osteoblast-like cells (MG63, ECACC, item code: 86051601) were cultured in conventional tissue culture flasks (250 mL, Greiner, Frickenhausen, Germany) in a humidified atmosphere at 37°C and 5% CO_2_. Although osteosarcoma derived, MG63 cells exhibit features in common with human osteoblast precursors or poorly differentiated osteoblasts. Specifically, these cells produce type I collagen with no or low basal osteocalcin (OC) and ALP [18]. However, when MG63 cells are treated with 1,25D, cell proliferation is inhibited and there is an accompanying, enhanced expression of OC. When MG63 cells are co-stimulated with 1,25D and selected growth factors, e.g., LPAs, then there is a marked, synergistic increase in ALP reflecting transition to the mature phenotype. Thus, the application of these cells to assess the compatibility of LPA-functionalised biomaterials for orthopaedic applications is entirely appropriate.

MG63 cells were grown to confluence in Dulbecco’s modified Eagle medium (DMEM)/F12 nutrient mix (Gibco, Paisley, Scotland) supplemented with sodium pyruvate (1 mM final concentration), L-glutamine (4 mM), streptomycin (100 ng/mL), penicillin (0.1 units/mL) and 10% v/v foetal calf serum (Gibco, Paisley, Scotland). The growth media was also supplemented with 1% of a 100x stock of non-essential amino acids. Once confluent, MG63 cells were subsequently dispensed into 24-well plates (Greiner, Frickenhausen, Germany) containing control and 16:0 OMPT functionalised Ti surfaces. In each case, wells were seeded with 1 mL of a 4x10^4^ cells/mL suspension (as assessed by haemocytometry) in serum free medium supplemented with 100 nM 1,25D. Cells were then cultured for 3 days, the Ti specimens removed and transferred to clean plates to ascertain cell number and total ALP activity for cells associated with the metal samples.

### Enumeration of MG63 cells

An assessment of cell number was performed using a combination of the tetrazolium compound 3-(4,5-dimethylthiazol-2-yl)-5-(3-carboxymethoxy-phenyl)-2-(4-sulfophenyl)-2H-tetrazolium, innersalt (MTS, Promega, UK) and the electron-coupling reagent phenazine methosulphate (PMS). Each compound was prepared separately in pre-warmed (37°C) phenol red-free DMEM/F12, allowed to dissolve, and then combined so that 1 mL of a 1 mg/mL solution of PMS was combined to 19 mL of a 2 mg/mL solution of MTS. A stock suspension of MG63 cells (1x10^6^ cells/mL) was serially diluted in growth medium to give a series of known cell concentrations down to 2.5x10^4^ cells/mL. Each sample (0.5 mL in a microcentrifuge tube) was spiked with 0.1 mL of the MTS/PMS reagent mixture and left for 45 minutes within a tissue culture incubator. Once incubated, the samples were centrifuged at 900 rpm to pellet the cells and 0.1 mL of the supernatants dispensed onto a 96-well microtitre plate and the absorbance read at 492 nm using a multiplate reader. Plotting the absorbances against known cell number, as assessed initially using haemocytometry, enabled extrapolation of cell numbers for the experiments described herein.

### Total alkaline phosphatase activity

An assessment of ALP activity is reliably measured by the generation of p-nitrophenol (p-NP) from p-nitrophenylphosphate (p-NPP) under alkaline conditions. The treatment of cells to quantify ALP activity was similar to that described by us recently [19]. Briefly, the MTS/PMS reagent was removed and the monolayers incubated for a further 15 minutes in fresh phenol red-free DMEM/F12 to remove the residual formazan. Following this incubation period, the medium was removed and the monolayers lysed with 0.1 mL of 25 mM sodium carbonate (pH 10.3), 0.1% (v/v) Triton X-100. After 2 minutes, each well was treated with 0.2 mL of 15 mM p-NPP (di-Tris salt, Sigma, UK) in 250 mM sodium carbonate (pH 10.3), 1 mM MgCl_2_. Lysates were then left under conventional cell culturing conditions for 1 h. After the incubation period, 0.1 mL aliquots were transferred to 96-well microtitre plates and the absorbance read at 405 nm. An ascending series of p-NP (25–500 micromolar) prepared in the incubation buffer enabled quantification of product formation. Unless stated otherwise, total ALP activity is expressed as the mean micromolar concentration of p-NP per 100,000 cells, as extrapolated from the MTS/PMS assay described above.

### Growth kinetics of *S*. *aureus*

Unless stated otherwise all media components used for growth of bacteria were from Sigma. Bacterial growth of the Methicillin Resistant strain *S*. *aureus* 43484 [20] was investigated in a 96 well microtiter plate. A single colony of strain MRSA 43484 from an overnight LB plate was inoculated into Luria Broth (LB) media and grown until exponential phase (around 4 hours) at 37°C and shaking of 200 rpm. Bacteria were then diluted 100 times in LB or minimal media as outlined and added calcium chloride when described. Minimal media consisted of M9 minimal media containing 2% glycerol, 0.5% cas-amino acids, 0.2 microgram/mL niacin, and 0.2 microgram/mL thiamine, and 2 mM magnesium chloride. EDTA, 16:0 OMPT and 16:0 MPPA were dissolved and diluted in PBS in two times the final concentration in a 96 well plate. Then the diluted compounds were mixed with equal amounts of the bacterial culture in the relevant media (LB or minimal media). Absorbance at OD 450 nm of the 96 well microtiter plate was read every 20 minutes the following 15 hours using a BMG Labtech FLUOstar OPTIMA Microplate Reader running at 37°C.

### Flow cytometry

A single colony of MRSA 43484 was inoculated into LB. After 4 hours incubation, this culture was used to inoculate LB media containing either 16:0 OMPT, 16:0 MPPA or EDTA at the concentrations specified or control, and incubated over night at 37°C, shaking at 200 rpm. Bacterial cultures were then washed in PBS (GIBCO, Grand Island, NY) and adjusted to approximately OD 600 nm 0,002. The bacterial suspension were then stained with 500nM propidium iodide for five minutes at 30°C before analysis on an Accuri C6 standard set-up flow cytometer (Accuri, Ann Arbor, MI). Initial gating of bacteria was performed using forward scatter (FSC-H) and side scatter (SSC-H). The gated population was further analysed using excitation of PI at 488 nm and emission at 670/LP nm (FL-3). The flow cytometer was operated at the Slow Flow Rate setting (14 microliter sample/minute).

### Bacterial adhesion to functionalised Ti

A single colony of MRSA 43484 was inoculated into LB media and grown overnight to stationary phase at 37°C and shaking (200 rpm). The culture was then washed once in LB and diluted to OD 600 nm 0,010 (corresponding to approximately 3x10^7^ CFU pr. mL) with fresh LB. 1 mL pr. well of the bacterial suspension was added to the wells of a 24 well plate (Nunc, Roskilde, Denmark) and Ti discs were added to the wells. The 24 well plate with Ti discs and bacteria was incubated for 8 h at 37°C. The Ti discs were then washed briefly in PBS and transferred to new wells with sterile PBS to loosen adhering bacteria. The plate was then stored at 4°C for 24–72 hours. The Ti discs were removed and CFU pr. mL of the detached bacteria (in PBS) was determined by spot plating (in duplicate). Four discs per group (control, OMPT or MPPA) were used and the experiment performed four times. To determine whether the coating on the Ti discs remained their activity after one time use, in one experiment the discs were reused: after the experiment the slides rinsed with soap and distilled water, padded dry and finally autoclaved and used as described above. During the rinsing procedure, mixing of the Ti discs was avoided by using separate glass vials for the different types of surface modification.

### Scanning electron microscopy

Bacterial adhesion was performed as described above, but instead of detaching bacteria by incubation in PBS, the briefly rinsed Ti discs with adhering bacteria were fixed in 2% glutaraldehyde in PBS, and postfixed in 1% OsO4. Samples were then dehydrated in ethanol, critical point-dried using CO_2_, and sputter-coated with gold. Samples were investigated using a Philips XL Feg30 scanning electron microscope operated at 2 kV. The number of adherent bacteria pr. micrometer^2^ was determined by manual counting of resulting images. Three images captured from different, random locations at the Ti disc were counted for each condition. For each sample the number of bacteria adhering to at least 10000 micrometer^2^ was determined. The SEM experiment was performed once.

### Statistical analyses

Unless stated otherwise, all the cell culture experiments described above were performed three times and all data were subject to a one-way analysis of variance (ANOVA) to test for statistical significance as we have reported previously [5]. When a p value of < 0.05 was found, a Tukey multiple comparisons post-test was performed between all groups. All data are expressed as the mean together with the standard deviation. Investigations of adhesion of MRSA to Ti discs were also performed at least three times unless otherwise stated and data were analysed with a one-way ANOVA using Dunnett’s multiple comparison post-test between control and treatment groups (functionalised Ti discs) for p values < 0.05.

## Results

### 3.1 16:0 OMPT and 1,25D co-operate to promote human osteoblast maturation

**Fig 2:**
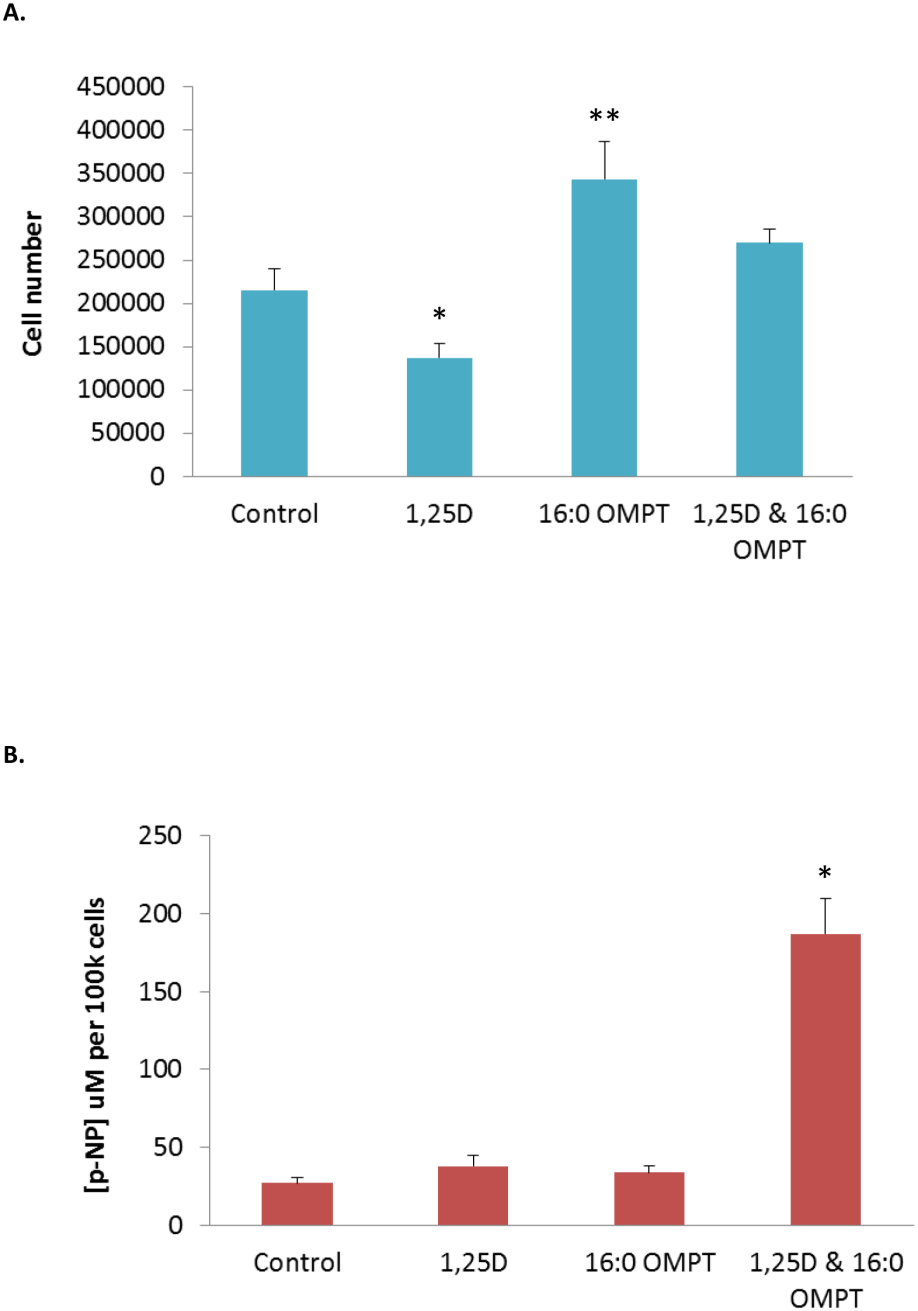
Growth and maturation of human (MG63) osteoblasts in response to 16:0 OMPT and 1,25D. **A.** MG63 osteoblasts were treated with 1,25D (100nM), 16:0 OMPT (micromolarμM) or a combination of these agents and left for three days under conventional cell culturing conditions prior to an assessment of cell growth. As anticipated for the pro-differentiating effects of 1,25D, there were fewer cells (*p<0.01 1,25D versus control) at the end of the culture period. In contrast the application of 16:0 OMPT led to a significant increase in cell numbers (**p<0.01 16:0 OMPT versus control). **B.** Alkaline phosphatase (ALP) is expressed in greater abundance as osteoblasts progress from an immature to a more differentiated phenotype. Enzyme activity is reliably monitored via the hydrolysis of p-nitrophenyl phosphate to p-nitrophenol (p-NP). The co-stimulation of MG63 cells with 100nM 1,25D and 16:0 OMPT yielded significant increases in total ALP activity by 72 h (*p < 0.001 versus agents in isolation). In each case the data shown are the mean + SD of 4 replicate samples and are a representative from three independent experiments.

Cell number increased significantly in the presence of 16:0 OMPT (10 micromolar), while 1,25D (100 nM) caused a significant reduction in cell number. However, the combined effect of 16:0 OMPT and 1,25D gave cell numbers similar to untreated cells (Fig 2A). After three days of culture, the extent of maturation of the osteoblasts in response to the active vitamin D_3_ metabolite; 1,25D and 16:0 OMPT was determined by measurement of alkaline phosphatase activity via quantification of p-NP from p-NPP. Co-stimulation of MG63 osteoblasts with 1,25D and 16:0 OMPT led to clear, synergistic, increases in ALP activity, as supported by elevated p-NP production (Fig 2B).

### 16:0 OMPT-functionalised Ti enhances 1,25D-induced MG63 maturation

**Fig 3:**
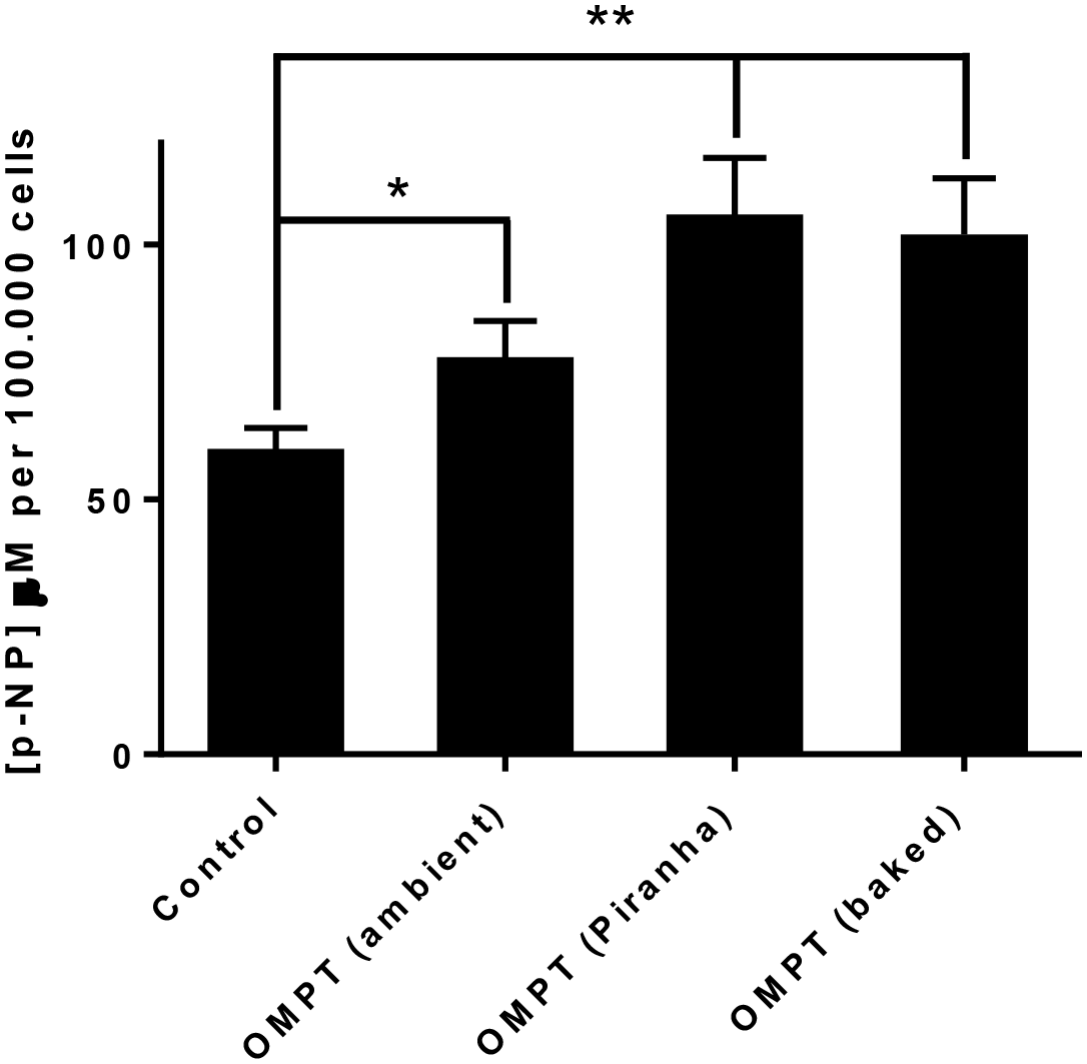
16:0 OMPT-functionalised Ti enhances 1,25D-induced MG63 cell maturation. Solid Ti discs (48 in total) were equally divided as follows: some were subjected to either Piranha solution (OMPT Piranha) or baked at 160°C overnight (OMPT baked) prior to steeping in ODPA for an hour under ambient conditions. Recovered samples were then exposed to 16:0 OMPT (0.1mM in 50% aqueous ethanol) overnight. Some discs were treated with ODPA alone (control) and others ODPA followed by 16:0 OMPT with no prior baking or Piranha treatment (OMPT ambient). Each group of discs were transferred to multiwell tissue culture plates and seeded with MG63 osteoblasts in a serum-free culture medium supplemented with 100nM 1,25D. After three days of culture an assessment of total ALP activity was performed using p-nitrophenylphosphate as substrate and quantification of p-nitrophenol (p-NP). The concentration of p-NP for cells upon ODPA/16:0 OMPT-functionalised Ti is greater than for cells on control Ti (*p<0.01 compared to control Ti). The extent of maturation, as supported by raised p-NP, is significantly greater still (**p<0.001 compared to control Ti) by prior baking/Piranha treatment of the Ti samples. The data presented are a representative of three independent experiments. In each case the data shown are the mean micromolar concentration of p-NP normalised to 10^5^ cells + SD of 4 replicate samples.

Human MG63 osteoblasts were seeded onto ODPA or ODPA-16:0 OMPT modified Ti specimens. Ti specimens subjected to ODPA-16:0 OMPT functionalisation caused a significantly higher degree of 1,25D-induced osteoblast maturation than ODPA alone (Fig 3). Moreover, pretreatment of the Ti specimens before surface modification either by baking or Piranha treatment gave even higher levels of p-NP (**p<0.001 versus ODPA control Ti) and thus MG63 osteoblast maturation than direct surface modification (16:0 OMPT ambient, *p<0.01 versus ODPA control Ti). No difference was found between baking and Piranha pretreatment with regard to MG63 maturation. Baking as pre-treatment option was used in all subsequent modifications for the microbiological studies described in the following.

### Antibacterial effects of 16:0 MPPA, 16:0 OMPT and EDTA

**Fig 4:**
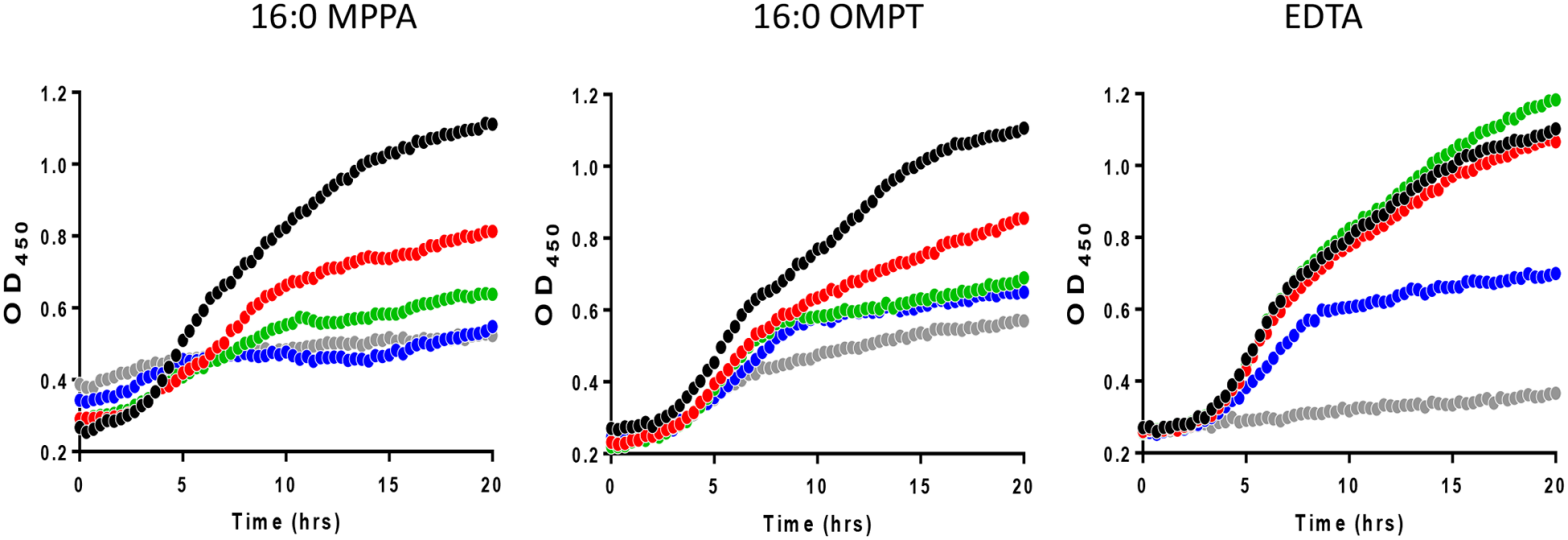
Lysophosphatidic acid species inhibit the growth of MRSA in Luria broth. The effect of different concentrations of 16:0 MPPA, 16:0 OMPT and EDTA on growth of MRSA 43484 in Luria broth. Legend: 16:0 MPPA; black (control), red (1,56 microgram/mL), green (6,3 microgram/mL), blue (25 microgram/mL). 16:0 OMPT; black (control), red (1,56 microgram/mL), green (6,3 microgram/mL), blue (25 microgram/mL). EDTA; black (control), green (6,3 microgram/mL), blue (25 microgram/mL), grey (0.1mg/mL).

**Fig 5:**
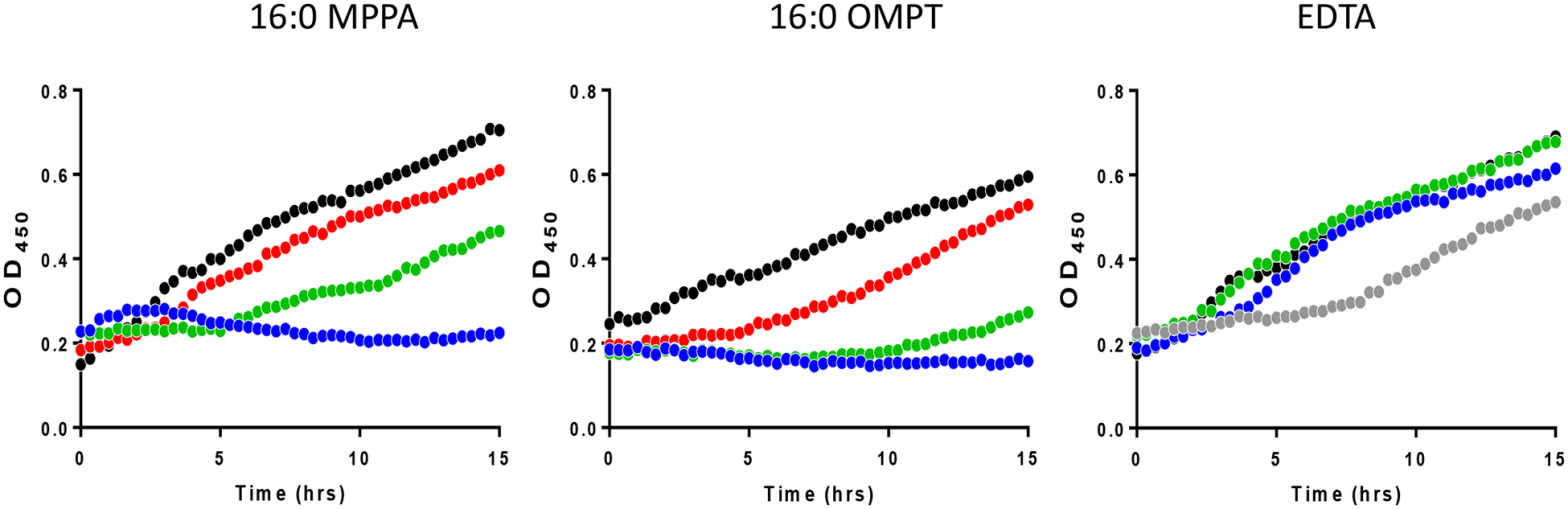
Lysophosphatidic acid species inhibit the growth of MRSA in minimal media. The effect of different concentrations of 16:0 MPPA, 16:0 OMPT and EDTA on growth of MRSA 43484 in minimal media. Legend: 16:0 MPPA; black (control), red (1,56 microgram/mL), green (6,3 microgram/mL), blue (25 microgram/mL). 16:0 OMPT; black (control), red (1,56 microgram/mL), green (6,3 microgram/mL), blue (25 microgram/mL). Notice that the symbols of the control almost hides the symbols of 6,3 microgram/mL EDTA.

**Fig 6:**
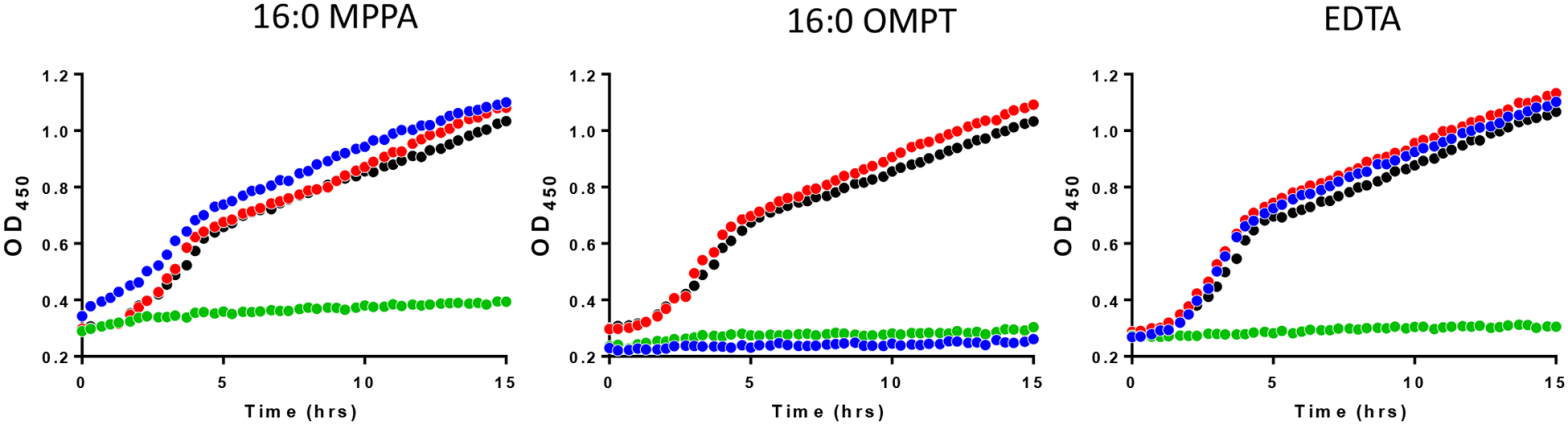
Growth of MRSA in response to lysophosphatidic acids and the influence of calcium. Growth curves of MRSA 43484 in minimal media showing the effect of Ca^2+^. Black (control), red (control + 2 mM Ca^2+^), green (100 μg/mL 16:0 MPPA/16:0 OMPT/EDTA), Blue 0.1mg/mL 16:0 MPPA/16:0 OMPT/EDTA + 2 mM Ca^2+^).

We have previously shown that 16:0 MPPA is antibacterial against a range of bacteria [11]. In order to investigate whether the artificial LPA analogue 16:0 OMPT had similar properties we exposed the MRSA clinical strain 43484 to serial dilutions of 16:0 OMPT and 16:0 MPPA and observed growth. We also included the chelator EDTA. Fig 4 shows that 200 μg/mL of 16:0 MPPA, 16:0 OMPT or EDTA (corresponding to 0.4 mM MPPA or OMPT or 0.5 mM EDTA) completely abolished growth of the MRSA strain. We found that the MRSA strain was much more sensitive to 16:0 MPPA and 16:0 OMPT in minimal media (Fig 5), where 25 microgram/mL of either 16:0 MPPA or 16:0 OMPT was enough to inhibit growth. We further investigated the effect of media composition. Fig 6 shows that addition of 2 mM Ca^2+^ abolished the growth inhibitory effect of 16:0 MPPA and EDTA while 16:0 OMPT still inhibited growth in the presence of extra calcium.

### Flow cytometry

**Fig 7:**
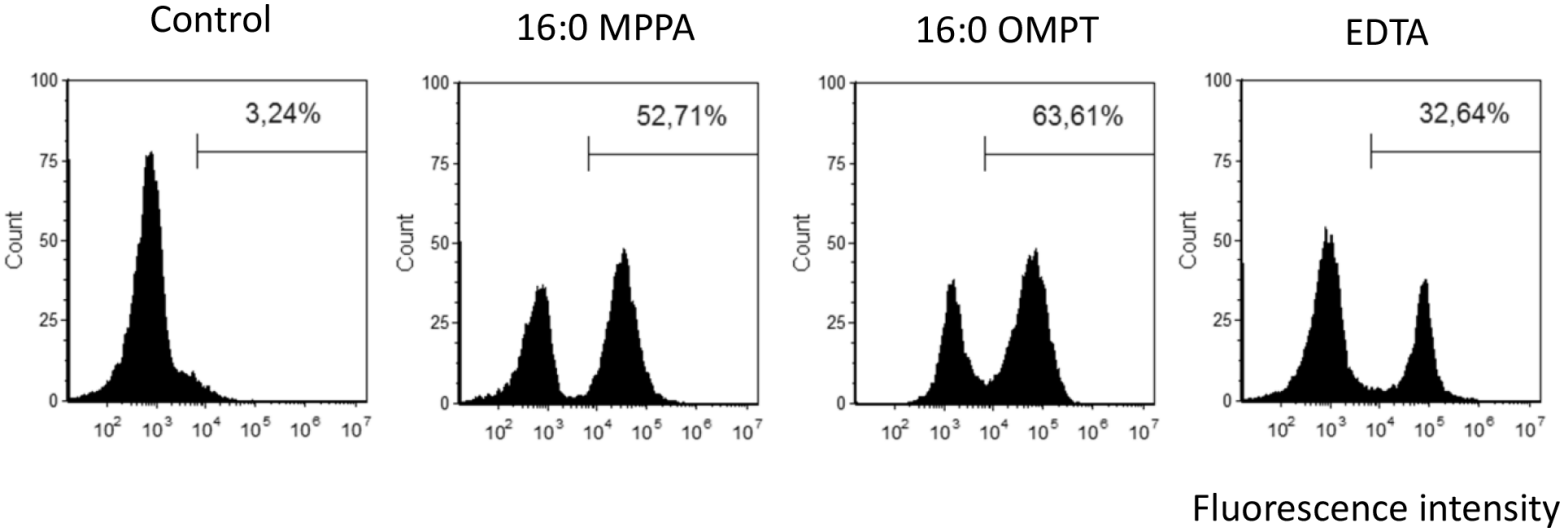
Flow cytometry of MRSA stained with PI: effect of lysophosphatidic acid species and EDTA on viability. Histograms of MRSA 43484 stained with propidium iodide (PI), gated on forward and side scatter. Bacteria were grown in the presence of 0.1mg/mL 16:0 MPPA, 50 microgram/mL 16:0 OMPT, 0.1mg/mL EDTA or in the absence of compounds (control). Markers show the percentage of PI stained, and hence nonviable, population.

To investigate whether 16:0 OMPT and 16:0 MPPA were bactericidal or bacteriostatic we investigated the uptake of the nonviable dye propidium iodide (PI) by MRSA grown in the presence of these compounds. The concentration of MPPA, OMPT and EDTA were adjusted to achieve limited growth; that is OD_600_ 1.5 compared to untreated bacteria that achieved an OD_600_ of 3.0. Fig 7 shows that the three compounds in the concentrations tested caused an increased uptake of PI, suggesting all three compounds to be bactericidal.

### Adhesion of MRSA to 16:0 OMPT and 16:0 MPPA functionalised Ti discs

**Fig 8:**
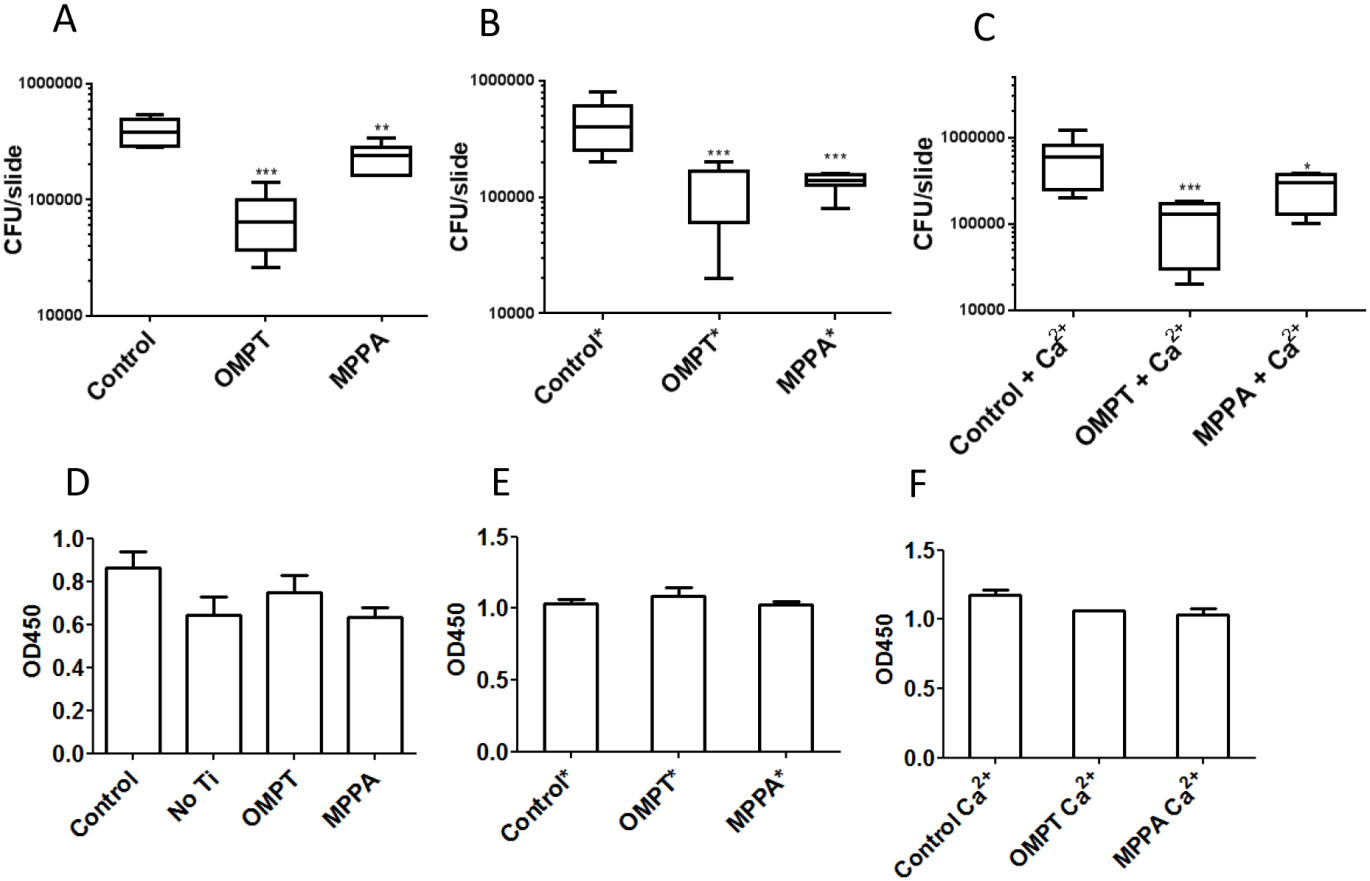
Lysophosphatidiate-functionalised titanium inhibits MRSA adhesion. Effect of functionalised Ti on bacterial adhesion. A-C) CFU of adherent MRSA pr. Ti disc after detachment. A) CFU pr. Ti disc; control or functionalised with either 16:0 OMPT or 16:0 MPPA. B) *Reuse of the same discs as in A) after washing and autoclaving. C) Effect of addition of 2 mM Ca^2+^ on MRSA adhesion to Ti discs. In each instance four discs were used for each of the different Ti surface treatments. D-F) End point OD_450_ of planktonic bacteria in the wells containing the Ti discs in A-C. (*p<0.05, **p<0.005 and ***p<0.0005 compared to control). Each of the figures is a representative of four independent experiments.

Aiming to apply the 16:0 OMPT and 16:0 MPPA to functionalise Ti for implant use we investigated the adhesion of MRSA strain 43484 to 16:0 MPPA or 16:0 OMPT functionalised Ti discs. Fig 8A shows that both 16:0 MPPA and 16:0 OMPT reduced adhesion of MRSA to Ti discs. Functionalisation using a linker such as ODPA should make the binding of compounds to the surface very durable through interaction of the hydrophobic alkyl chains of both the ODPA and the LPA/LPA analogue. To test this, we reused the Ti discs in a new experiment after careful rinsing and autoclaving of the Ti discs. Fig 8B shows that in the reuse experiment the anti-adhesive effect reached the same level as the first round of the experiment. As mentioned above we found that adding Ca^2+^ abolished the growth inhibitory effect of 16:0 MPPA, and we wanted to investigate whether extra Ca^2+^ would also affect the anti-adhesive effect of 16:0 MPPA and/or 16:0 OMPT. Fig 8C shows that adding 2 mM Ca^2+^ did not affect the anti-adhesive properties of 16:0 MPPA or 16:0 OMPT functionalisation. Fig 8D–8G shows that growth in the media above the functionalised Ti discs (Fig 8A–8C) were not affected, suggesting that low/no 16:0 MPPA or 16:0 OMPT was released from the Ti discs into the media.

### Scanning Electron Microscopy

**Fig 9:**
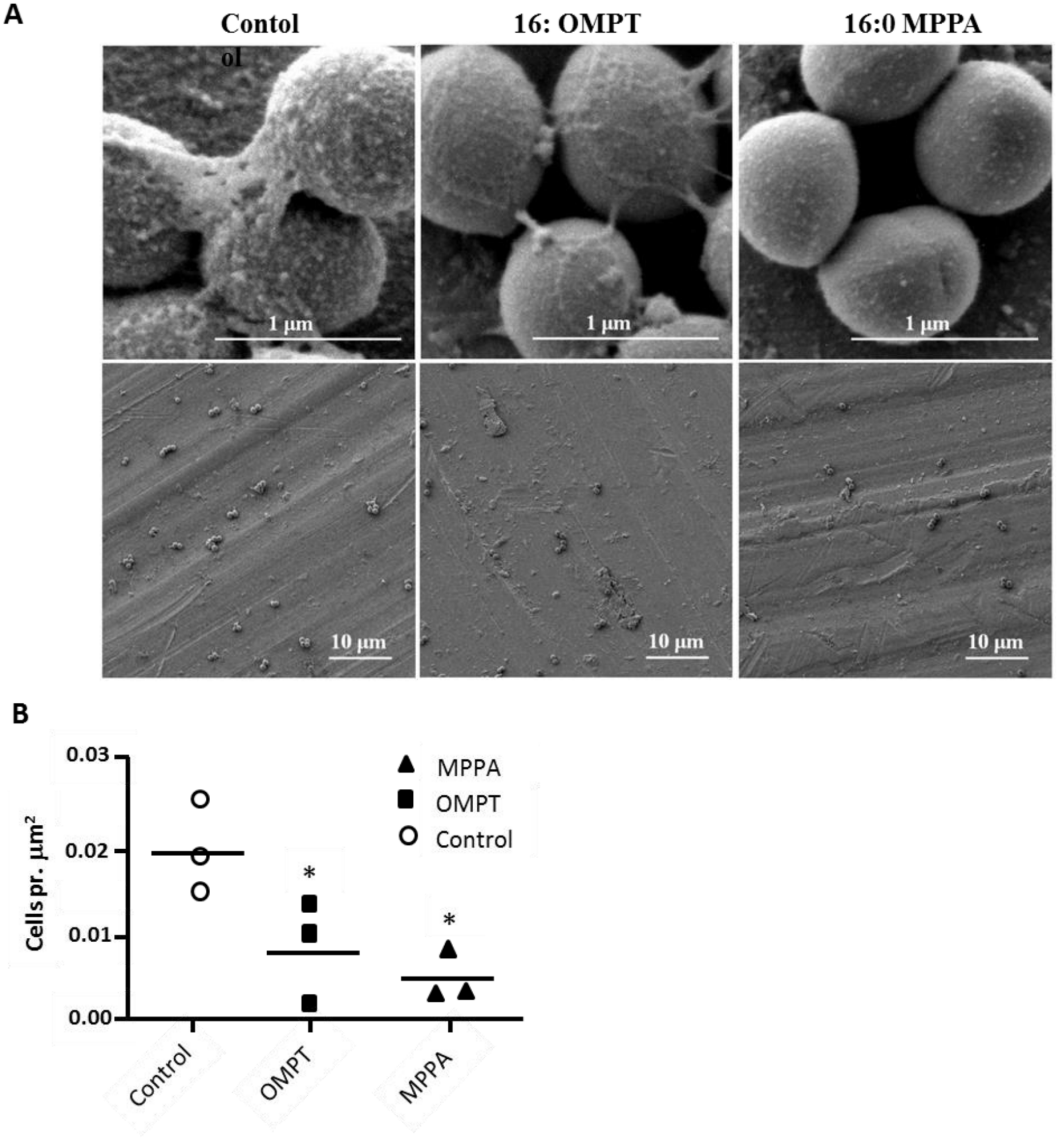
Lysophosphatidate-functionalisation of titanium deters bacterial attachment and alters bacterial surface morphology. **A.** Scanning Electron Microscopy images of MRSA 43484 on control and functionalised Ti surfaces. The left column shows unmodified Ti, in the centre, Ti functionalised with 16:0 OMPT, and in the right column images of bacteria upon 16:0 MPPA functionalised Ti. **B.** Graph depicting the data obtained for the number of bacteria associated for each of the different Ti surfaces.

Scanning Electron Microscopy (SEM) was performed on functionalised Ti discs with adherent MRSA bacteria. Examples of resulting images are shown in Fig 9. Manual counting of adherent bacteria/micrometerm^2^ showed that significantly fewer bacteria adhered to the 16:0 OMPT and 16:0 MPPA functionalised Ti compared to the control. Interestingly, the images showed that while the bacteria adhering to the control Ti discs had a very rough appearance, bacteria adhering the 16:0 OMPT and especially the 16:0 MPPA functionalised Ti discs appeared smooth and with less extracellular matrix material.

## Discussion

Developing novel biomaterial coatings that enhance early osseointegration yet deter the attachment of bacteria are especially desirable properties for future orthopaedic and dental implant devices. The simple pleiotropic lipid, LPA and/or selected LPA analogues, could represent candidate molecules for coating Ti. Over a decade ago Laux and colleagues [9] described how 16:0 MPPA (palmitoyl LPA) inhibited the growth and virulence of *P*. *aeruginosa*. 16:0 MPPA is a naturally occurring LPA found in biological fluids including bronchoalveolar lavage (BAL), serum and plasma [21–23]. Interestingly, exposure of bacteria to this lipid made them increasingly susceptible to certain antibiotics. This particular lipid species sits amongst several structurally similar lysophospholipids that serve as signalling molecules to most mammalian cell types [24] In the context of human osteoblast biology LPAs co-operate with 1,25D to enhance their maturation [3,4] an event synonymous with bone tissue formation. Given the reported effects of 16:0 MPPA on bacteria suggest LPA/LPA analogues as potential adjuncts in a bone regenerative setting and one way in which this could be realised is by coating biomaterials with LPA/LPA analogues. Such a “dual-action” coating would be particularly appealing to reduce potential infection risk of implantable devices whilst encouraging superior early osseointegration.

Human osteoblasts and bone marrow stromal cells express at least three different LPA receptor types. When stimulated with LPA/LPA analogues effects ranging from proliferation, fibronectin binding, cytoskeletal reorganization and differentiation have been reported [2]. In this particular investigation we found that 16:0 OMPT stimulated MG63 cell growth. When the same cells were co-stimulated with 1,25D and 16:0 OMPT cell growth was modestly attenuated and there was a synergistic increase in total ALP activity indicating a change towards a more mature or differentiated phenotype. All of these changes essentially mirror the effects we have seen for other LPA species on this cell type. In this study we also show that the functionalisation of Ti with 16:0 OMPT, enhances 1,25D-induced MG63 maturation, as supported by the greater total ALP activity. The effect of 16:0 OMPT functionalisation on maturation of MG63 is particularly encouraging; mature osteoblasts are responsible for bone matrix synthesis and mineralisation and if these events can be enhanced at the Ti surface then this could facilitate the process of early osseointegration.

We have previously shown that 16:0 MPPA sensitises otherwise betalactam resistant *P*. *aeruginosa* to the actions of ampicillin [9,10]. We have also shown that this LPA is antibacterial against a number of Gram positive bacteria including *S*. *aureus* [11]. Here we further show that the phosphatase resistant LPA analogue, 16:0 OMPT, is antibacterial against MRSA strain 43484. The mechanism by which the two compounds exert their effects is unclear. We have previously reported that 16:0 MPPA precipitates in the presence of the divalent cations calcium and magnesium and that precipitation is most noticeable with the former ion. These observations imply that 16:0 MPPA can function as a chelator [10]. Due to the clear structural similarities between 16:0 MPPA and 16:0 OMPT, it is likely that 16:0 OMPT is also capable of forming chelating micelles in the presence of divalent cations. In 1968 it was reported that EDTA reversed ampicillin resistance in *P*. *aeruginosa*; the minimum inhibitory concentrations (MIC) of ampicillin was reduced from 0.5mg/mL to less than 2 microgram/mL in the presence of 3.4 mM EDTA [25]. The lipopolysaccharide (LPS) layer in Gram negative bacteria such as *P*. *aeruginosa* are stabilised by divalent cations (mainly Ca^2+^). If calcium ions are removed by treatment with a chelator, LPS is liberated, and through this disruption the membrane becomes more permeable to other agents, causing a potentiating action (reviewed in [26]). Instead of LPS, Gram positive bacteria have surface sugars, phosphate groups and basic residues organised as teichoic acid. *S*. *aureus* teichoic acids are composed of alternating phosphate and ribitol or glycerol groups, and modified with D-alanine and N-acetylglucosamine. Teichoic acids are important for binding of Mg^2+^ to the *S*. *aureus* cell wall [27] and possibly other divalent cations such as Ca^2+^. The cell surface of *S*. *aureus* has a moderately negative net charge at neutral pH, which is probably due to the fact that the teichoic acid contain less positively charged D-alanine residues than negatively charged [28]. The charge of teichoic acid has been reported to be important for primary adhesion of bacteria to a substrate surface [29], which is the initial step of biofilm formation. Moreover, the negative charge have been reported to play a role for resistance against glycopeptide antibiotics [30]. It is thus possible that the chelating ability of 16:0 OMPT and 16:0 MPPA is important for their antibacterial activity through membrane destabilisation. On the other hand, chelators could also cause simple growth inhibition by depriving the bacteria of essential cations. To investigate this we performed flow cytometry analyses (Fig 7), which revealed that a high proportion of the cultures treated with 16:0 MPPA and 16:0 OMPT stained positive with PI suggesting that the two compounds directly kill the bacteria, and not just reduce proliferation. Interestingly we found that the while the effect on growth of 16:0 MPPA and EDTA was abolished by addition of 2 mM Ca^2+^, 16:0 OMPT still inhibited growth in the presence of extra calcium (Fig 6).

The antibacterial and osteoblast maturation enhancing activity of the LPA (analogue) compounds lead us to investigate bacterial adhesion to 16:0 MPPA and 16:0 OMPT functionalised Ti discs. *S*. *aureus* adhesion to the functionalised Ti discs were significantly reduced, however, growth in the media above the functionalised Ti discs were not affected (Fig 8). Moreover, the antiadhesive effect was not reduced in experiments with reuse of functionalised Ti discs. Thus, it seems that only very low or no 16:0 MPPA or 16:0 OMPT were released from the Ti discs into the media. As we found that addition of extra calcium abolished the growth inhibitory effect of 16:0 MPPA, we also investigated the effect of adding additional calcium to the media in which the functionalised Ti discs were placed. However the extra added calcium did not alter bacterial adhesion to neither 16:0 MPPA nor 16:0 OMPT-functionalised surfaces.

We also performed SEM on functionalised Ti discs with adherent MRSA bacteria. In accordance with findings obtained using the CFU method, we found that significantly fewer bacteria adhered to the 16:0 OMPT and 16:0 MPPA functionalised Ti compared to the control. The SEM images revealed an interesting and clear difference in morphology between the bacteria adhering to the control, which had a very uneven and coarse surface, and bacteria adhering the 16:0 OMPT and especially the 16:0 MPPA functionalised Ti discs, which appeared smooth and with less extracellular matrix material. This suggests that 16:0 OMPT and 16:0 MPPA affect the teichoic acid layer, destabilising the bacterial outer membrane and thereby affecting both surface attachment and viability.

Whilst our findings offer a novel route to the biological modification of Ti we are cognisant that considerably more research will be required to optimise the surface coating. In addition steps to ascertain coating stability and robustness will be priority areas in realising the application of this technology for bone regenerative applications. Specifically an assessment of coating persistence to washing, sterilisation and the physical forces encountered during implantation will need to be part of a wider research plan prior to any *in vivo* evaluation in the future.

## Conclusion

To summarise we present evidence for the successful bio-functionalisation of Ti with 16:0 MPPA/OMPT by adopting the facile pre-attachment of an APA via the natural oxide layer of Ti. This surface finish exhibited two noteworthy properties that may help realise the application of selected LPAs in future bone biomaterial design; in the first instance the surfaces enhanced 1,25D-induced hOB maturation. In addition this same surface deterred the attachment of *S*.*aureus* and there were noticeable differences in bacterial surface morphology pointing to the possible loss of teichoic acid. Collectively our findings point towards the development of a “dual action” Ti device of which similar technologies have yet to be reported.

## Abbreviations

1,25D: 1,25-dihydroxy vitamin D_3_
ALP: alkaline phosphatase
APAs: alkane phosphonic acids
BMSC: bone marrow-derived stromal cell
hOB: human osteoblast
LPAs: lysophosphatidic acids
16:0 MPPA: monopalmitoyl phosphatidic acid (also known as 1-palmitoyl-2-hydroxy-*sn*-glycero-3-phosphate)
MTS: 3-(4,5-dimethylthiazol-2-yl)-5-(3-carboxymethoxy-phenyl)-2-(4-sulfophenyl)-2H-tetrazolium
OC: osteocalcin
16:0 OMPT: O-2-(hexadecyloxy)-3-methoxypropyl O-hydrogenphosphorothioate
PMS: phenazine methosulphate
Ti: Titanium
TJR: total joint replacement
