# Correction: Dual Action of Lysophosphatidate-Functionalised Titanium: Interactions with Human (MG63) Osteoblasts and Methicillin Resistant *Staphylococcus aureus*

**DOI:** 10.1371/journal.pone.0147276

**Published:** 2016-01-21

**Authors:** Mette Elena Skindersoe, Karen A. Krogfelt, Ashley Blom, Jianxing Zhang, Guowei Jiang, Glenn D. Prestwich, Jason Peter Mansell

Dr. Jianxing Zhang should be included in the author byline. He should be listed as the fourth author, and his affiliation #6: Department of Medicinal Chemistry, The University of Utah, Salt Lake City, Utah, United States of America. The contributions of this author are as follows: Conception the design of the work, acquisition and analysis/interpretation of data, synthesized the molecules employed in the experiments, contributed to reagents/materials/analysis tools, drafting as well as revising of the manuscript, and final approval of the published version.

The correction citation is: Skindersoe ME, Krogfelt KA, Blom A, Zhang J, Jiang G, Prestwich GD, et al. (2015) Dual Action of Lysophosphatidate-Functionalised Titanium: Interactions with Human (MG63) Osteoblasts and Methicillin Resistant *Staphylococcus aureus*. PLoS ONE 10(11): e0143509. doi:10.1371/journal.pone.0143509
